# Supplementary material for: GATA6 is predicted to regulate DNA methylation in an in vitro model of human hepatocyte differentiation
Source: Commun Biol. 2022 May 4;5:414. doi: 10.1038/s42003-022-03365-1 (PMC9068788; doi:10.1038/s42003-022-03365-1)
Supplement: Supplementary file 2 — Supplementary Infomation [file 42003_2022_3365_MOESM2_ESM.pdf]

Supplementary Figure 1: Expression of differentiation marker and DNA methylation-related genes

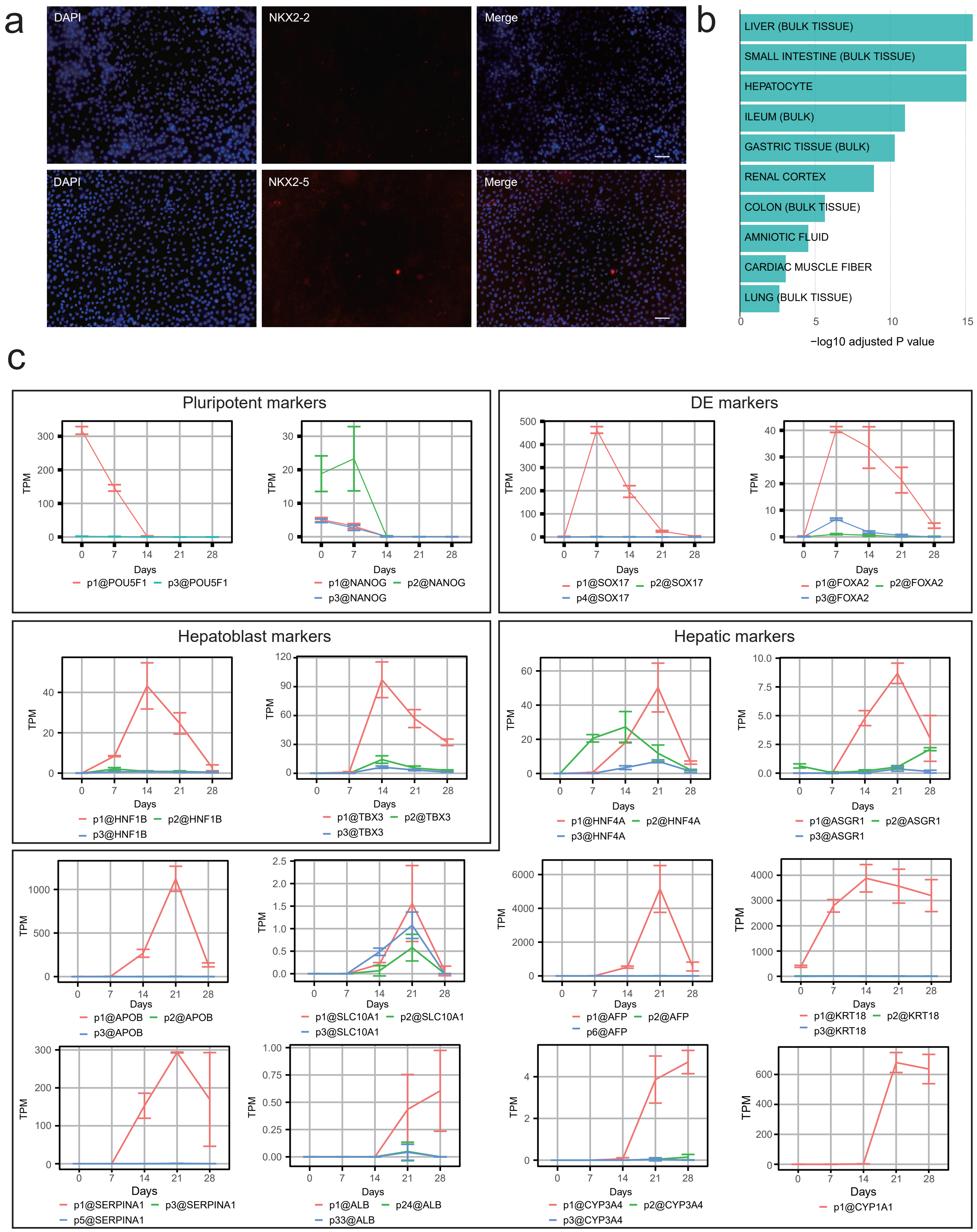

(a)Immunocytochemistry for NKX2.2 and NKX2.5 in day 21 cells. The scale bar is 50 µm. (b) Tissue-specific gene enrichment analysis. The bar blot shows enrichment of top 10 specifically expressing tissues of the upregulated genes on day 28 compared with day 0. The X-axis represents −log10 adjusted P-value. (c) Line plot showing average TPM of each promoter of pluripotent, DE, and hepatic differentiation marker genes. The error bar is the standard deviation. X- and Y-axis are days of differentiation and average TPM, respectively. The experiment was performed in three biological replicates.

Supplementary Figure 2: AFP secretion and DNA methylation-related gene expressions.

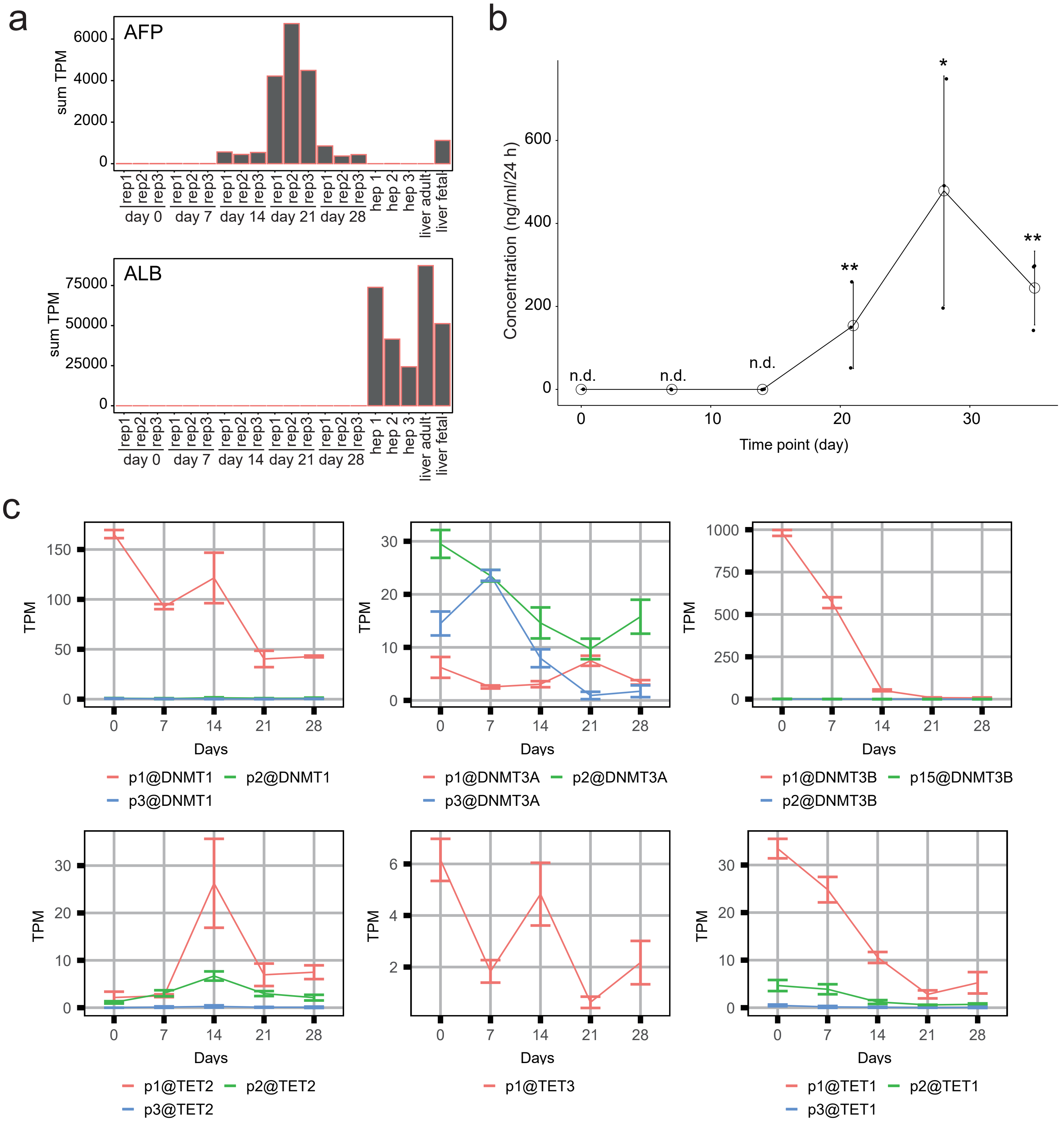

(a) AFP and ALB CAGE expression data of in vitro hepatic differentiation time-course and published primary hepatocyte, adult liver, and fetal liver (Forrest. A. et al. Nature 507, 462-470 (2014)). Replicate number is abbreviated to the rep, hep 1: hepatocyte donor 1, hep 2: hepatocyte donor 2, hep 3: hepatocyte donor 3. (b) AFP levels in culture medium. The medium was replaced with fresh medium three days before the collection. The X-axis is the days of differentiation. Y-axis is the concentration per 24 hours. Each open circle and error bar are mean and standard deviation. Black dots are individual data point. n.d. is "not detected" showing below the detection limit. The experiment was performed in four biological replicates with two technical replicates (*$p < 0.05$; **$p < 0.01$; One-sided Student's t-test). (c) Line plot showing average TPM of each promoter of DNA methylation-related genes. The error bar is the standard deviation. X- and Y-axis are days of differentiation and average TPM, respectively. The experiment was performed in three biological replicates.

Supplementary Figure 3: Enriched Gene Ontologies at differentially methylated regions.

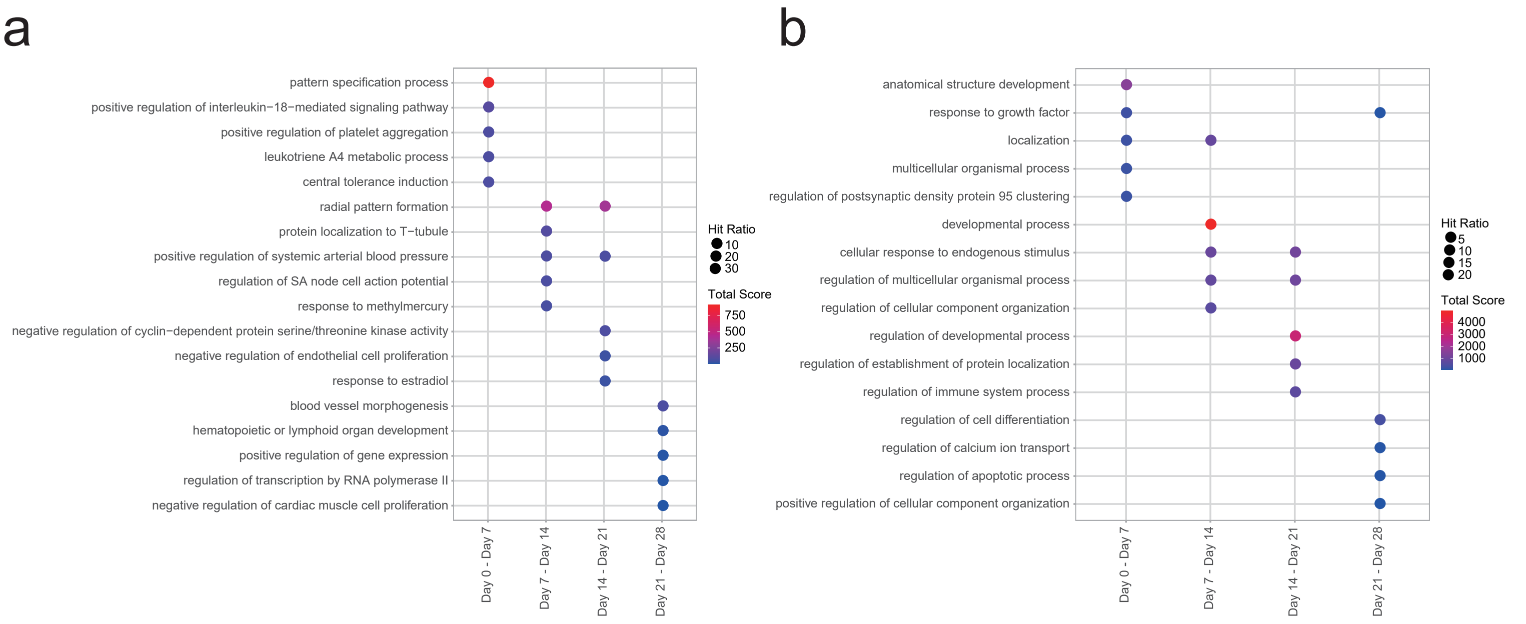

Top 5 enriched summarized GOs of methylated (a) and demethylated (b) regions. The dot size represents the ratio between the number of probes that hit the GOs and all methylated or demethylated probes. The color represents total scores computed by reduceSimMatrix function of the rrvgo R package.

Supplementary Figure 4: Expression profile of putative DNA methylation-regulating TFs

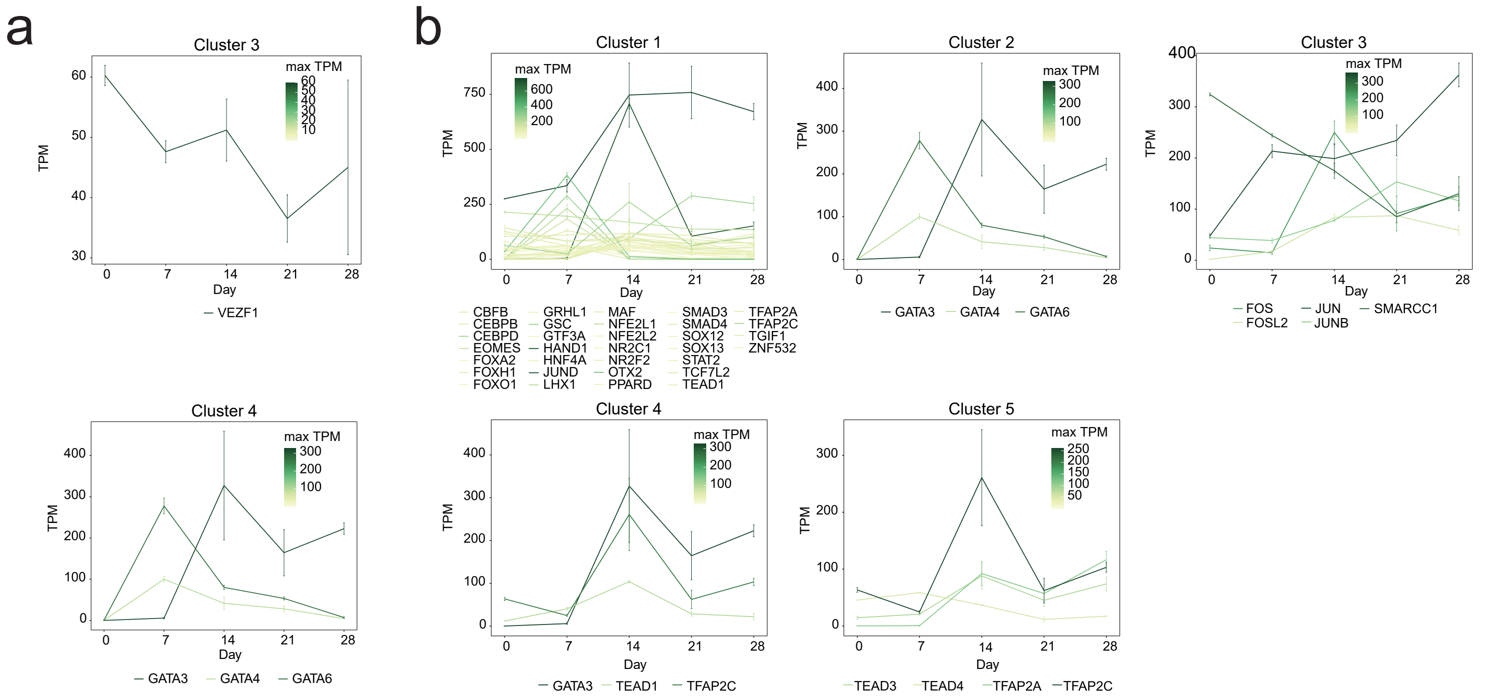

mRNA expression profile of the genes corresponding to the overrepresented TF binding motifs at methylated (a) and demethylated (b) regions. X- and Y-axes show time points of differentiation (hours from differentiation initiation) and tag-per-million (TPM) of CAGE, respectively. The color of each line and the error bar represents the maximum TPM and s.d, respectively. The experiment was performed in three biological replicates.

Supplementary Figure 5: DNA demethylation by GATA6 overexpression.

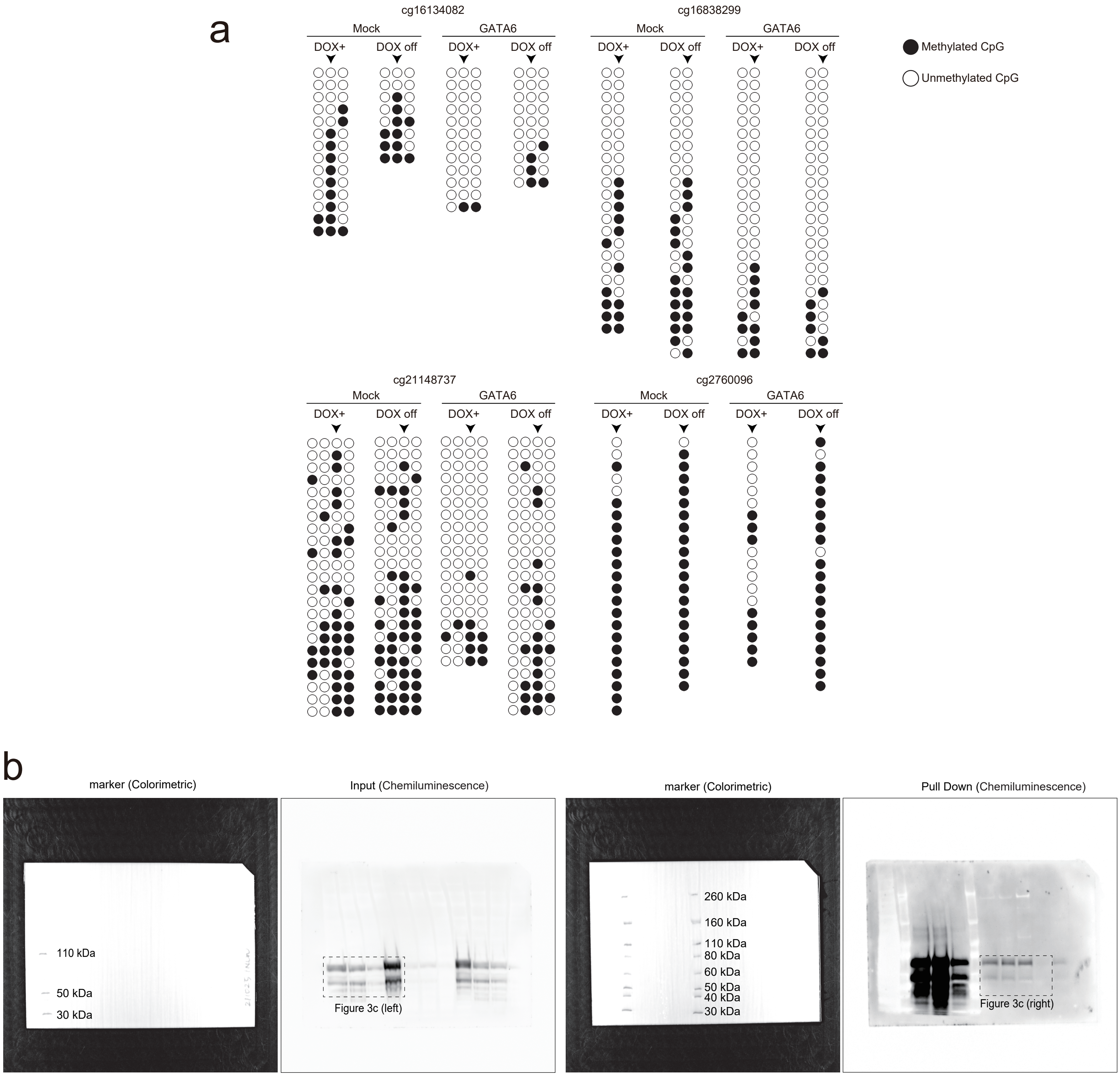

(a) Cloning-based bisulfite sequencing for GATA6 overexpression and withdrawing. DNA methylation patterns of ±100 bp regions from four selected demethylated regions, analyzed by cloning-based bisulfite sequencing. Black and white circles indicate methylated and unmethylated cytosine of CpG, respectively. Horizontal lines represent the sequencing result of each sub-clone. Arrowheads indicate the CpGs that are demethylated in methylation array analysis. (b) Uncropped unprocessed western blot of Fig. 3c.

Supplementary Figure 6: Transcriptome and methylome analysis of iPS cell to DE differentiation.

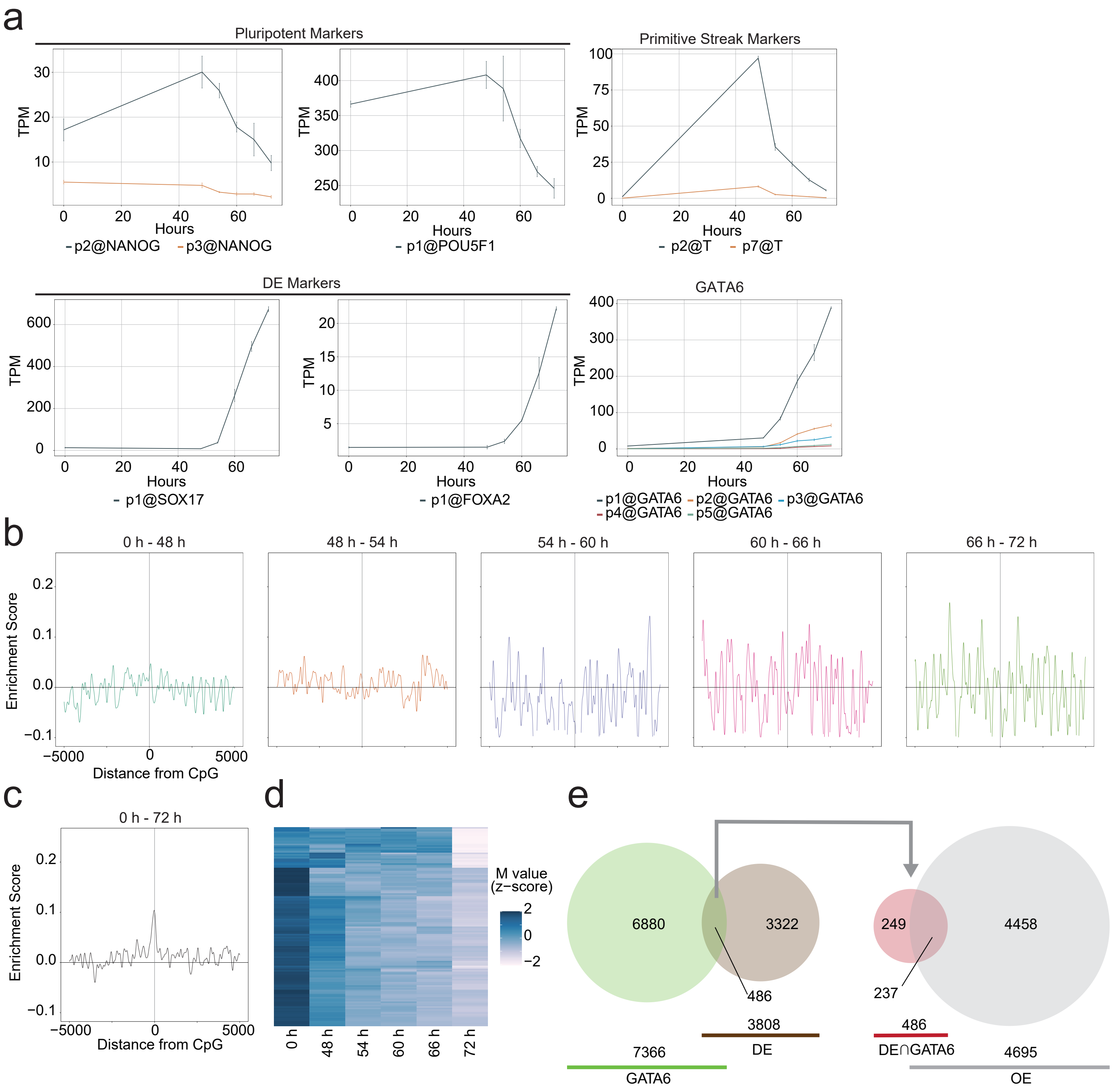

(a) Line plot showing average TPM of each promoter of the pluripotent, primitive streak, and DE differentiation marker genes. The error bar is the standard deviation. X- and Y-axis are days of differentiation and average TPM, respectively. The experiment was done in triplicate. (b, c) Distribution of enrichment score for the GATA6 binding motif within ±5,000 bp of demethylated CpG probes at each interval of adjacent timepoint (b) and 0 h vs. 72 h (c). X- and Y-axes show distance from probe CpG position and enrichment score, respectively. Horizontal and vertical lines are enrichment score = 0 and demethylated CpG position, respectively. The colors of each plot represent colors of timepoints shown in Fig. 4A. (d) A heatmap showing the M-value at each timepoint of the probes demethylated in 72 h compared with 0 h. The color represents the z-scored M-value. (e) Venn diagrams showing the overlap between GATA6 ChIPmentation peaks (GATA6) and demethylated regions of the DE differentiation stage (DE) (left) and the overlap between the intersect regions of the left Venn diagram (DE∩GATA6) and demethylated regions induced by GATA6 overexpression in 293T cells (OE) (Miyajima et al. Chromosome Res. (2022)).

Supplementary Figure 7: Chromatin status at UDRs.

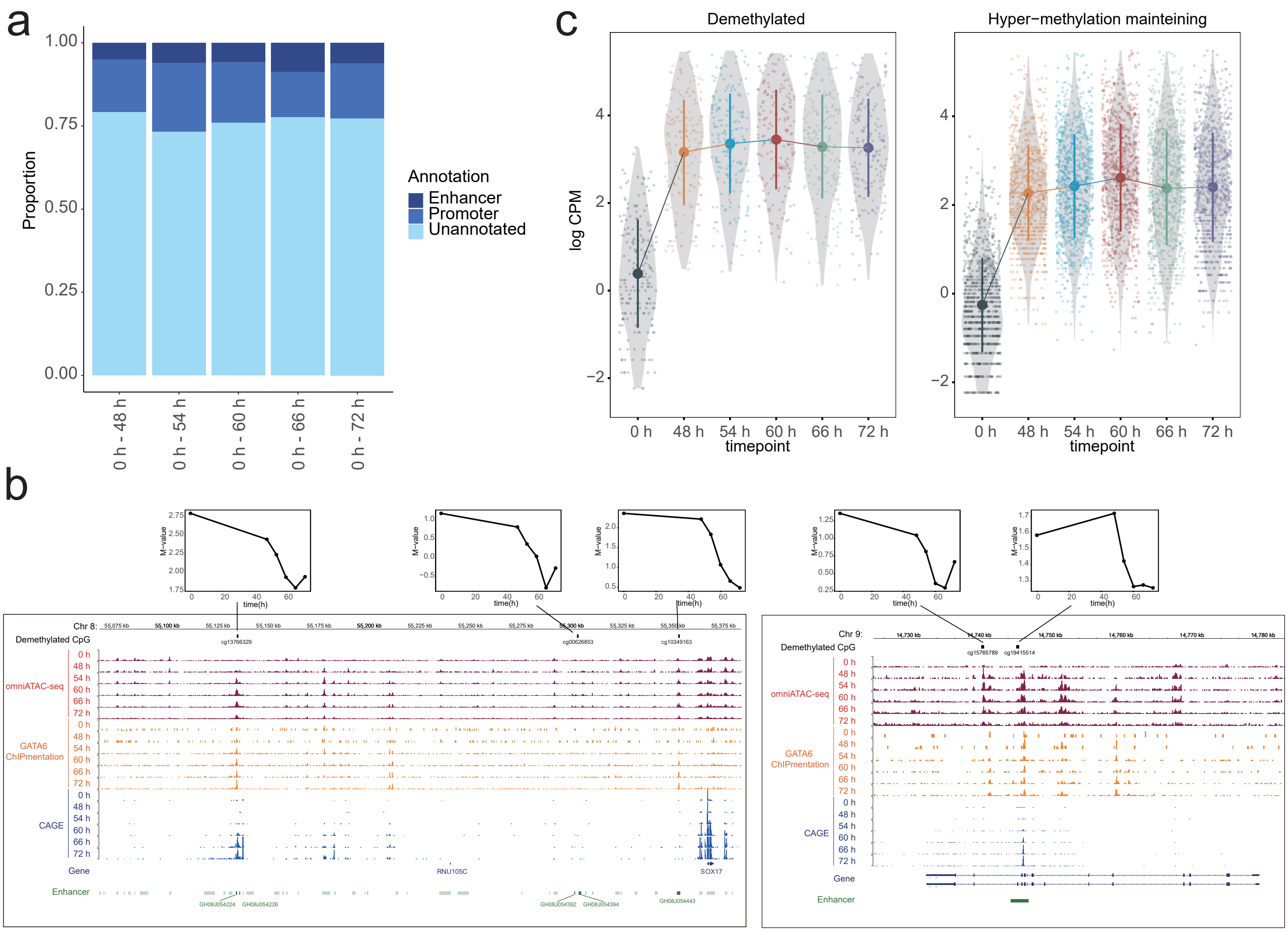

(a)The proportion of UDRs by regulatory region annotation. X- and Y-axis represent comparison and proportion, respectively. (b) Representative screenshots of the genome viewer. DNA demethylated regions shown in the genome viewer are GATA6 ChIPmentation read coverage and OmniATAC-read coverage at the upstream region of SOX17 and the FREM1 locus. The scale of each dataset is coverage of 10 million 100 nt reads. Red translucent rectangles represent demethylated regions. Enhancer track is based on the GeneHancer database, and enhancers overlapped with the demethylated region are shown as dark green. The genome version is hg19. M-value profiles of each demethylated probe are shown above (x-axis: time point (hour), y-axis: M-value). (c) Violin plots showing chromatin accessibility of ATAC-seq peaks opened in the period 0 to 48 h with the demethylated regions and hyper-methylation-maintaining regions. X- and Y-axis are DE differentiation timepoints and log Count Per Million (CPM). Vertical bar, fill circle located at the center of the vertical bar, and gray shade at each data represent s.d, mean, and distribution of data. Open circle represents each data point.

Supplementary Table 1 Primer List

qRT-PCR

Gene Symbol	Forward Primer	Reverse Primer
GATA6	GAGCCCCTACTCGCCCTAC	GACAGGTCCTCCAGCAGGT
GATA4	GCTCCTACTCCAGCCCCTAC	GTGGACATAGCCCCACAGTT

Cloning-based bisulfite sequencing

Target Probe ID	Forward Primer	Reverse Primer
cg16134082	TATTTAGTGGGTTGTGATTTAGGAG	AAATAATCAAAACAACATATTTCCATATAA
cg16838299	GAAATATAGAGGAATTAAAAGTTAGTATTA	ACCTCCATTTTATAAATAAAAAAAC
cg21148737	GGTAAATAATGAAAATTTTTAGGGTG	CTTCTTCCTTCAACACAAAAACTAC
cg27160096	TGAATAGGTTTTGGGGTGTAATATAAT	AATCAAACAATCAAAACAATCCAATA

Supplementary Table 2 Antibody List

Antibody	Manufacture	Cat #	Lot #	Dilution
**Immunocytechemistry**				
anti-HNF4a	abcam	ab92378	GR3339194-1	1:100
anti-Nkx2.2	abcam	ab191077	GR3173859-8	1:50
anti-Nkx2.5	Cell Signaling Technology	8792	3	1:400
anti-AFP	Takara bio	M225	AK301	1:100
anti-Rabbit IgG (H+L) alexafluor 594 conjugated	ThremoFisher Scientific	A32740	UD286650	1:200
anti mouse IgG alexafluor 488 conjugated	ThremoFisher Scientific	A21202	2018296	1:200
**ChIPmentation**				
anti-GATA6 antibody (D61E4)	Cell Signaling Technology	5851S	4	1:50
**HaloTag pull-down assay**				
anti-hGATA6	R & D	AF1700	KWT0421021	1:1000
VeriBlot for IP secondary antibody	abcam	ab131366	GR190998-5	1:5000
