# Supplementary material for: GATA6 is predicted to regulate DNA methylation in an in vitro model of human hepatocyte differentiation
Source: Commun Biol. 2022 May 4;5:414. doi: 10.1038/s42003-022-03365-1 (PMC9068788; doi:10.1038/s42003-022-03365-1)
Supplement: Supplementary file 3 — Description of Additional Supplementary Files [file 42003_2022_3365_MOESM3_ESM.pdf]

Description of Additional Supplementary Files

File name: Supplementary Data 1
Description: Source data underlying main figures.
