# Supplementary material for: GATA6 is predicted to regulate DNA methylation in an in vitro model of human hepatocyte differentiation
Source: Commun Biol. 2022 May 4;5:414. doi: 10.1038/s42003-022-03365-1 (PMC9068788; doi:10.1038/s42003-022-03365-1)
Supplement: Supplementary file 5 — Reporting Summary [file 42003_2022_3365_MOESM5_ESM.pdf]

nature research

Corresponding author(s): Harukazu Suzuki

Last updated by author(s): Apr 13, 2022

Reporting Summary

Nature Research wishes to improve the reproducibility of the work that we publish. This form provides structure for consistency and transparency in reporting. For further information on Nature Research policies, see our Editorial Policies and the Editorial Policy Checklist.

Statistics

For all statistical analyses, confirm that the following items are present in the figure legend, table legend, main text, or Methods section.

n/a	Confirmed	
☐	☒	The exact sample size (*n*) for each experimental group/condition, given as a discrete number and unit of measurement
☐	☒	A statement on whether measurements were taken from distinct samples or whether the same sample was measured repeatedly
☐	☒	The statistical test(s) used AND whether they are one- or two-sided<br>*Only common tests should be described solely by name; describe more complex techniques in the Methods section.*
☐	☒	A description of all covariates tested
☐	☒	A description of any assumptions or corrections, such as tests of normality and adjustment for multiple comparisons
☐	☒	A full description of the statistical parameters including central tendency (e.g. means) or other basic estimates (e.g. regression coefficient) AND variation (e.g. standard deviation) or associated estimates of uncertainty (e.g. confidence intervals)
☐	☒	For null hypothesis testing, the test statistic (e.g. F, t, r) with confidence intervals, effect sizes, degrees of freedom and P value noted<br>*Give P values as exact values whenever suitable.*
☒	☐	For Bayesian analysis, information on the choice of priors and Markov chain Monte Carlo settings
☐	☒	For hierarchical and complex designs, identification of the appropriate level for tests and full reporting of outcomes
☐	☒	Estimates of effect sizes (e.g. Cohen's *d*, Pearson's *r*), indicating how they were calculated

Our web collection on statistics for biologists contains articles on many of the points above.

Software and code

Policy information about availability of computer code

Data collection	For CAGE, the data were acquired using the Hiseq 2500 sequencer.<br>For ATAC-seq, the data were acquired using the Hiseq 2500 sequencer.<br>For ChIPmentation, the data were acquired by Microgen, Inc using the Hiseq X10 sequencer.<br>For Methylation array, the data were acquired using Illumina iScan syste.
Data analysis	A description of the software and code has been included in the Methods.

For manuscripts utilizing custom algorithms or software that are central to the research but not yet described in published literature, software must be made available to editors and reviewers. We strongly encourage code deposition in a community repository (e.g. GitHub). See the Nature Research guidelines for submitting code & software for further information.

Data

Policy information about availability of data

All manuscripts must include a data availability statement. This statement should provide the following information, where applicable:

- Accession codes, unique identifiers, or web links for publicly available datasets
- A list of figures that have associated raw data
- A description of any restrictions on data availability

The datasets generated and analyzed during the current study are available in the NCBI Gene Expression Omnibus (GEO; http://www.ncbi.nlm.nih.gov/geo/) under accession number GSE1633310. The reviewers can access to the data with the token ahgzcesuhhstxup.
R scripts generated for the analysis are available on GitHub (https://github.com/RIKEN-CFCT/Hep_methylation_TF).

Field-specific reporting

Please select the one below that is the best fit for your research. If you are not sure, read the appropriate sections before making your selection.

☒ Life sciences ☐ Behavioural & social sciences ☐ Ecological, evolutionary & environmental sciences

For a reference copy of the document with all sections, see nature.com/documents/nr-reporting-summary-flat.pdf

Life sciences study design

All studies must disclose on these points even when the disclosure is negative.

Sample size	For methylation array analyses, n=1 was chosen as the replicate number because we discussed overall tendency such as the transcription factor motif enrichment but not each individual DMR, which is sufficient for the analyses.<br>For CAGE, n=3 was chosen as the replicate number. The consistently very high correlation between these replicates , suggests that this is sufficient.<br>For ChIPmentation and omniATAC-seq analyses, n=2 was chosen as the replicate number. The consistently very high correlation between these replicates, suggests that this is sufficient.
Data exclusions	No data were excluded
Replication	Although each time-course methylation array analyses performed n=1, enriched motifs at demethylated regions duriong definitive endoderm differentiations were reproduced.<br>For CAGE, ChIPmentation, and ominiATAC-seq, the correlations between replicates were very high, indicating the high reproducibility of the experiments.
Randomization	No randomization in this study.
Blinding	The investigators were not blinded during data collection.

Reporting for specific materials, systems and methods

We require information from authors about some types of materials, experimental systems and methods used in many studies. Here, indicate whether each material, system or method listed is relevant to your study. If you are not sure if a list item applies to your research, read the appropriate section before selecting a response.

Materials & experimental systems

n/a	Involved in the study	
☐	☒	Antibodies
☐	☒	Eukaryotic cell lines
☒	☐	Palaeontology and archaeology
☒	☐	Animals and other organisms
☒	☐	Human research participants
☒	☐	Clinical data
☒	☐	Dual use research of concern

Methods

n/a	Involved in the study	
☐	☒	ChIP-seq
☒	☐	Flow cytometry
☒	☐	MRI-based neuroimaging

Antibodies

Antibodies used	anti-GATA6 antibody (clone: D61E4, cat no.5851S : lot:4, Cell Signaling Technology, Inc.)<br>anti-HNF4a antibody(Cat no.: ab92378, lot:GR3339194-1 abcam)<br>anti-Nkx2.2 antibody (Cat no.: ab191077, lot:GR3173859-8, abcam)<br>anti-Nkx2.5 antibody (Cat no.: 8792, lot:3, Cell Signaling Technology Inc.)<br>anti-AFP antibody (Cat. no.: M225, lot:AK301, Takara bio)<br>anti-Rabbit IgG (H+L) alexafluor 594 conjugated (Cat.no.: A32740, lot:UD286650, ThremoFisher Scientific)<br>anti mouse IgG alexafluor 488 conjugated (Cat.no.: A21202, lot:2018296, ThremoFisher Scientific)
Validation	anti-GATA6 antibody: https://www.cellsignal.jp/products/primary-antibodies/gata-6-d61e4-xp-rabbit-mab/5851<br>anti-HNF4a antibody: https://www.abcam.co.jp/nkx22-antibody-epr14638-n-terminal-ab191077.html<br>Anti-Nkx2.2 antibody: https://www.abcam.co.jp/nkx22-antibody-epr14638-n-terminal-ab191077.html<br>Anti-Nkx2.5 antibody: https://www.cellsignal.jp/products/primary-antibodies/nkx2-5-e1y8h-rabbit-mab/8792<br>anti-AFP antibody: https://catalog.takara-bio.co.jp/com/tech_info_detail.php?mode=3&masterid=M100005783&unitid=U100003019<br>anti-Rabbit IgG (H+L) alexafluor 594 conjugated: https://www.thermofisher.com/antibody/product/Goat-anti-Rabbit-IgG-H-L-Highly-Cross-Adsorbed-Secondary-Antibody-Polyclonal/A32740<br>anti mouse IgG alexafluor 488 conjugated: https://www.thermofisher.com/antibody/product/A-21202.html?gclid=CjwKCAiAyPyQBhB6EiwAFUuaks0DkEIE1azwplJTU21YbYbJCC0CyEWjD-

WGEF5I1tufXW4iy2QDGBoCOQkQAvD_BwE&ef_id=CjwKCAiAyPyQBhB6EiwAFUuaks0DkEIE1azwplJTU21YbYbJCC0CyEWjD-WGEF5I1tufXW4iy2QDGBoCOQkQAvD_BwE:G:s&s_kwcid=AL!3652!3!516608152455!!!g!!&cid=bid_pca_aus_r01_co_cp1359_pjt0000_bid00000_0se_gaw_dy_pur_con

Eukaryotic cell lines

Policy information about cell lines

Cell line source(s)	The 201B7 human iPS cell line and HEK293T (RCB2202) cell line from RIKEN bio resource center (BRC)
Authentication	Non of the cell lines used were authenticated .
Mycoplasma contamination	Cell lines were not tested for mycoplasma contamination.
Commonly misidentified lines (See ICLAC register)	No commonly misidentified cell lines were used.

ChIP-seq

Data deposition

☒ Confirm that both raw and final processed data have been deposited in a public database such as GEO.

☒ Confirm that you have deposited or provided access to graph files (e.g. BED files) for the called peaks.

Data access links
May remain private before publication.

The datasets generated and analyzed during the current study are available in the NCBI Gene Expression Omnibus (GEO; http://www.ncbi.nlm.nih.gov/geo/) under accession number GSE163331. The reviewers can access to the data with the token ahgzcesuhhstxup.

Files in database submission

GATA6_0h.sorted.bw
GATA6_48h.sorted.bw
GATA6_54h.sorted.bw
GATA6_60h.sorted.bw
GATA6_66h.sorted.bw
GATA6_72h.sorted.bw
GATA6_0h_1e-5_summits.bed
GATA6_48h_1e-5_summits.bed
GATA6_54h_1e-5_summits.bed
GATA6_60h_1e-5_summits.bed
GATA6_66h_1e-5_summits.bed
GATA6_72h_1e-5_summits.bed
DE_0h_rep1_R1.fastq.gz
DE_0h_rep1_R2.fastq.gz
DE_0h_rep2_R1.fastq.gz
DE_0h_rep2_R2.fastq.gz
DE_48h_rep1_R1.fastq.gz
DE_48h_rep1_R2.fastq.gz
DE_48h_rep2_R1.fastq.gz
DE_48h_rep2_R2.fastq.gz
DE_54h_rep1_R1.fastq.gz
DE_54h_rep1_R2.fastq.gz
DE_54h_rep2_R1.fastq.gz
DE_54h_rep2_R2.fastq.gz
DE_60h_rep1_R1.fastq.gz
DE_60h_rep1_R2.fastq.gz
DE_60h_rep2_R1.fastq.gz
DE_60h_rep2_R2.fastq.gz
DE_66h_rep1_R1.fastq.gz
DE_66h_rep1_R2.fastq.gz
DE_66h_rep2_R1.fastq.gz
DE_66h_rep2_R2.fastq.gz
DE_72h_rep1_R1.fastq.gz
DE_72h_rep1_R2.fastq.gz
DE_72h_rep2_R1.fastq.gz
DE_72h_rep2_R2.fastq.gz

Genome browser session (e.g. UCSC)

Mapped reads were visualized in IGV(https://tinyurl.com/yj3otxq7)

Methodology

Replicates	For ChIPmentation analyses, n=2 was chosen as the replicate number. The consistently very high correlation between these replicates, suggests that this is sufficient.

Sequencing depth	GATA6_0h.bam: 48055744 + 0 in total (QC-passed reads + QC-failed reads), 46036545 + 0 mapped (95.80% : N/A), 150bp paired-end<br>GATA6_48h.bam: 41133060 + 0 in total (QC-passed reads + QC-failed reads), 39667958 + 0 mapped (96.44% : N/A), 150bp paired-end<br>GATA6_54h.bam: 142169738 + 0 in total (QC-passed reads + QC-failed reads), 137756623 + 0 mapped (96.90% : N/A), 150bp paired-end<br>GATA6_60h.bam: 79302130 + 0 in total (QC-passed reads + QC-failed reads), 76787052 + 0 mapped (96.83% : N/A), 150bp paired-end<br>GATA6_66h.bam: 131170324 + 0 in total (QC-passed reads + QC-failed reads, 126555399 + 0 mapped (96.48% : N/A), 150bp paired-end<br>GATA6_72h.bam: 158406858 + 0 in total (QC-passed reads + QC-failed reads), 152941857 + 0 mapped (96.55% : N/A), 150bp paired-end
Antibodies	anti-GATA6 antibody (clone: D61E4, lot:5851S, Cell Signaling Technology, Inc.)
Peak calling parameters	p-value < 1e-10
Data quality	Ratio of significant peaks (Irreproducible discovery rate (IDR) < 0.05)<br>0 h: 42.9 % (GATA6 is not expressed)<br>48 h: 52.4 % (GATA6 is not expressed)<br>54 h: 8.7 %<br>60 h: 24.5 %<br>66 h: 0.6 %<br>72 h: 4.8 %
Software	The analysis code is available on GitHub (https://github.com/RIKEN-CFCT/Hep_methylation_TF)
